# Deficits in Emotion Recognition and Theory of Mind in Parkinson’s Disease Patients With and Without Cognitive Impairments

**DOI:** 10.3389/fpsyg.2022.866809

**Published:** 2022-05-13

**Authors:** Alessandra Dodich, Giulia Funghi, Claudia Meli, Maria Pennacchio, Chiara Longo, Maria Chiara Malaguti, Raffaella Di Giacopo, Francesca Zappini, Luca Turella, Costanza Papagno

**Affiliations:** ^1^Center for Mind/Brain Sciences CIMeC, University of Trento, Rovereto, Italy; ^2^Department of Psychology, University of Milano-Bicocca, Milan, Italy; ^3^Dipartimento di Scienze Neurologiche, Ospedale Santa Chiara, Trento, Italy; ^4^Azienda Provinciale per i Servizi Sanitari, Trento, Italy

**Keywords:** emotion recognition, theory of mind, Parkinson’s disease, mild cognitive impairment, social cognition

## Abstract

**Background:**

Emotion recognition and social deficits have been previously reported in Parkinson’s disease (PD). However, the extent of these impairments is still unclear and social cognition is excluded from the cognitive domains considered in the current criteria for PD mild cognitive impairment (MCI). This study aims to analyze emotion recognition, affective and cognitive theory of mind in early PD patients classified according to Level II MCI criteria, and to evaluate the prevalence of socio-cognitive deficits in this sample.

**Methods:**

We enrolled 45 participants with PD, classified as cognitively unimpaired (CU; *n* = 32) or MCI (*n* = 13) based on a standard neuropsychological assessment. Social cognitive skills were evaluated through validated tests for emotion recognition (i.e., Ekman 60-faces test, Ek60 Test) and mental states attribution (Story-based Empathy Task, SET) and compared to a group of 45 healthy controls (HC). Between-group differences in social tasks were performed, as well as correlation analyses to assess the relationship between social, cognitive, and clinical variables. Finally, the number of patients with social cognitive impairments in both MCI and CU subgroups was computed based on Italian normative data.

**Results:**

Statistical comparison revealed significant differences among groups in the Ek60 test, with MCI obtaining significantly lower scores than HC and CU, especially for negative emotions. Significant differences were detected also in the SET, with lower performance in emotion and intention attribution for both PD groups compared to HC. A significant correlation emerged between the Ek60 test and emotion attribution. Nine patients showed poor performance at social tasks, five of them being classified as PD-CU.

**Discussion:**

Parkinson’s disease cognitive profile was characterized by emotion recognition and attribution deficits. These results, as well as the detection of CU patients with isolated socio-cognitive impairments, underline the importance of assessing social cognition in PD as a possible early marker of cognitive decline.

## Introduction

Parkinson’s disease (PD) is a progressive multisystem neurodegenerative disorder (Dickson, 2018) characterized at the clinical level by a constellation of motor and non-motor symptoms (Trojano and Papagno, 2018), among which cognitive impairments have received particular attention due to their consequences in everyday functioning [e.g., Leroi et al. (2012)]. Alongside the well-known deficits in executive functioning, visuo-spatial abilities and memory, recent evidence has underlined possible socio-cognitive impairments in PD. Social cognition is a complex cognitive domain, which refers to a set of different processes aimed at recognizing and interpreting signals from the environment, understanding self and others’ behaviors, and adapting the response based on social needs (Frith, 2008). As a multi-faceted domain, social processes required for successful social interaction include aspects of social perception (e.g., emotion recognition), theory of mind (ToM, also defined as mental states attribution), empathy and social behavior (Henry et al., 2016). Notwithstanding some controversial results, deficits of social cognition in PD have been previously reported, with the majority of studies showing significant emotion recognition deficits, particularly for negative emotions (Gray and Tickle-Degnen, 2010; Argaud et al., 2018; Coundouris et al., 2019). Interestingly, these deficits seem to be at least partially independent from depressive symptomatology (Gray and Tickle-Degnen, 2010; De Risi et al., 2018), executive dysfunctions (Saenz et al., 2013; Enrici et al., 2015) or early visual processing deficits (Mattavelli et al., 2021). From a neuroanatomical perspective, emotion recognition disorders have been previously related to neurodegenerative alterations in regions belonging to the mesocorticolimbic pathway (Ibarretxe-Bilbao et al., 2009; Baggio et al., 2012), including the amygdala (Diederich et al., 2016). Considering its role as a hub within neural networks responsible for multisensorial and affective processing, damage to this brain region is hypothesized to hamper not only emotion recognition in PD, but also the attribution of affective mental states to others (i.e., affective ToM) (Premack and Woodruff, 1978; Call and Tomasello, 2008; Shamay-Tsoory et al., 2010; Diederich et al., 2016). Consistently, ToM dysfunctions have been recently described in PD patients (Bora et al., 2015), although the extent to which these deficits involve cognitive (i.e., ability to infer other intentions or beliefs) or affective (i.e., ability to infer other emotions) subcomponents is still an open issue, with some evidence supporting an early impairment in the cognitive sub-component and a later involvement of affective processing [e.g., Poletti and Bonuccelli (2012)], and other supporting early alterations in both ToM aspects [e.g., Santangelo et al. (2012)]. Besides, to the best of our knowledge, only a few studies investigated both emotion recognition and affective ToM in PD (Enrici et al., 2015; Foley et al., 2019; Alonso-Recio et al., 2021), and the relationship between these two facets, as well as the presence of these deficits in the earliest disease stages, is still unclear.

Despite the above-mentioned evidence of socio-cognitive deficits in PD, social cognition is not included among the cognitive domains clinically assessed for the definition of PD cognitive status according to the 2012 Movement Disorder Society (MDS) criteria for the detection of mild cognitive impairment (MCI) (Litvan et al., 2012). These criteria include a two-level operational schema depending on the neuropsychological assessment. Together with an abbreviated evaluation (i.e., MDS Level I criteria), specific guidelines for a comprehensive cognitive assessment (i.e., MDS Level II criteria) were defined. According to these recommendations, a patient is classified as MCI when showing impairments on at least two neuropsychological tests (i.e., one impaired test in two different cognitive domains or two impaired tests in one cognitive domain) in specific cognitive domains (i.e., attention and working memory, executive function, language, memory, and visuo-spatial functions). A recent study (Czernecki et al., 2021) evaluated the prevalence of socio-cognitive deficits in PD patients characterized by the MDS level I criteria. Notably, this study showed socio-cognitive dysfunctions in 30% of the sample, of which 20% was classified as cognitively unimpaired (CU). However, the narrow neuropsychological assessment, including only a measure of cognitive screening (i.e., MoCA) and a global measure of executive functioning (i.e., Frontal Behavioral Inventory) prevented a full MCI characterization (Litvan et al., 2012). In this study, we aim to fill this gap by investigating socio-cognitive deficits in PD patients characterized according to MDS Level II MCI criteria (Litvan et al., 2012). In order to analyse the prevalence of socio-cognitive dysfunctions in the clinical setting, we focused on tests validated for the Italian population assessing social perception (emotion recognition) and ToM.

## Materials and Methods

### Subjects

Forty-five patients with PD diagnosed according to the United Kingdom Parkinson’s Disease Society brain bank criteria (Hughes et al., 1992) were enrolled at the Center for Neurocognitive Rehabilitation of the Center for Mind/Brain Sciences (University of Trento) from January 2020 to November 2021. Inclusion criteria were a diagnosis of idiopathic PD, Hoehn and Yahr score ≤ 3 (Hoehn and Yahr, 1967), age above 50 years old and being under anti-parkinsonian medication. Patients with evidence of dementia or other neuropsychiatric disorders were excluded. All patients underwent a baseline clinical evaluation performed by experienced neurologists and neuropsychologists and were tested while in their medication-on condition. To evaluate the possible effect of clinical features, levodopa equivalent daily dose (LEDD) was determined and correlation analyses have been performed considering socio-cognitive performance, LEDD, disease duration and Hoehn and Yahr stage.

Forty-five healthy controls (HC), matched for demographic variables to the patient group, were also enrolled for statistical comparison at social tasks. HC were included based on the absence of positive neuropsychiatric history, neurological disorders, or cognitive impairment evaluated through the Italian version of the Montreal Cognitive Assessment (MoCA, cut-off score < 19.501) (Conti et al., 2015). The study was conducted in accordance with the ethical guidelines of the local ethics committee and the Declaration of Helsinki and written informed consent was signed by all participants.

### Neuropsychological Assessment

All patients underwent a standard neuropsychological evaluation including a test of global cognitive status (Conti et al., 2015), as well as two tests for each cognitive domain as suggested by MDS criteria (Litvan et al., 2012). Attentive matrices (Spinnler and Tognoni, 1987) and backward digit span (Monaco et al., 2013) were selected for attention and working memory, phonemic verbal fluency (Carlesimo et al., 1996), and Stroop task (Caffarra et al., 2002b) for executive functions, line orientation judgment test and unknown face recognition test (Benton, 1983) for visuo-spatial abilities, and two naming tasks for language (Catricalà et al., 2013; Papagno et al., 2020). Long-term memory was assessed through delayed recall at verbal [Rey Auditory Verbal Lists test–RAVLT (Carlesimo et al., 1996)] and non-verbal [Rey-Osterrieth Complex Figure ROCF (Caffarra et al., 2002a)] tests (see Table 1 for a summary of the neuropsychological tasks included in the assessment). Based on Italian normative cut-off scores, patients were classified as CU or MCI following the MDS Level II criteria (Litvan et al., 2012). PD-MCI patients were also classified into subtypes based on the presence of abnormalities on two tests within a single domain (single-domain PD-MCI) or multiple deficits in different cognitive domains (multi-domain PD-MCI). Finally, the presence of mood disorders was evaluated through the Geriatric Depression Scale (GDS) (Yesavage, 1988) and the Parkinson Anxiety Scale (Santangelo et al., 2016).

**TABLE 1:** Neuropsychological tests used for Parkinson’s disease (PD) profile classification according to PD-MCI Level II criteria.

Cognitive domain	Cognitive tests
Attention and working memory	• Attentive matrices • Digit span backward
Executive functions	• Phonemic verbal fluency task • Stroop task
Visuospatial abilities	• Benton’s judgment of line orientation • Benton facial recognition test
Long-term memory	• Rey auditory verbal list delayed recall • Rey-Osterrieth complex figure delayed recall
Language	• Naming of colored pictures • Naming of actions

### Socio-Cognitive Assessment

Socio-cognitive abilities have been evaluated through a test of emotion recognition (Ekman 60-faces Test—Ek60) (Dodich et al., 2014) and a test of mental state attribution (story-based empathy task—SET) (Dodich et al., 2015). The Ek60 is a well-known test used to assess emotion recognition abilities from static images expressing six basic emotions (i.e., fear, disgust, anger, happiness, sadness, surprise), shown on a computer monitor each for 5 seconds according to the Italian normative procedure. No time limit was set for patients’ responses. A global score, as well as scores for recognition of single emotions, can be computed. The maximum score is 60 for the whole test and 10 for each basic emotion. The SET is a non-verbal task developed to assess mental states attribution in neurodegenerative diseases associated with dementia (Cerami et al., 2015; Dodich et al., 2016; Dodich et al., 2021a; Valera-Bermejo et al., 2021), which has been also applied to other neurological populations (Realmuto et al., 2015; Campanella et al., 2021). This test includes a sub-test of emotion attribution (SET-EA), as well as a condition of intention attribution (SET-IA) and causal inference (SET-CI). Each condition has a sub-score of a maximum of six points, with a global score of 18 indicating the best possible performance.

### Statistical Analysis

First, preliminary statistical analyses were performed to evaluate data distribution (Shapiro-Wilk test) and to compare demographic variables (i.e., age, education, and sex) between groups (PD-CU, PD-MCI, HC) through one-way ANOVA and Chi-squared test.

Differences in basic neuropsychological tasks were evaluated between PD-MCI and PD-CU using *t*-student statistics or Mann-Whitney U based on data distribution.

Performances at social tasks (Ek60 Test and SET adjusted scores) were compared between PD-CU, PD-MCI, and HC using parametric or non-parametric (Kruskal-Wallis) one-way ANOVA. *Post-hoc* analyses were carried out through the Games-Howell test (for parametric statistics) and Dwass-Steel-Crotchlow-Fligner pairwise comparison (for non-parametric statistics). Since a significant difference between PD-CU and PD-MCI was found in education, analyses were performed on adjusted scores according to normative values. Then, partial correlation analyses controlling for global cognitive status (i.e., MoCA) were performed between the main neuropsychological variables of interest to assess the relationship between socio-cognitive abilities and clinical, cognitive and behavioral functioning in PD. Finally, we evaluated the number of patients with socio-cognitive impairments in both MCI and CU subgroups based on Italian normative cut-off values for Ek60 global score, affective (SET-EA) and cognitive (SET-IA) mental states attribution. Notably, we used the approach of “equivalent scores” proposed by Capitani and Laiacona (1997). This method allows mapping patients’ performance into an ordinal five-point scale (range 0–4), where “0” indicates a pathological performance, “1” a borderline performance, and “2–4” a normal performance. Statistical analyses were conducted using Jamovi 2 (Fox and Weisberg, 2020; Jamovi, 2021; R Core Team, 2021).

## Results

### Cognitive Profile in Parkinson’s Disease Patients

According to level II PD-MCI criteria, 32 patients classified as PD-CU and 13 patients as PD-MCI (Table 2). All PD-MCI were classified as multiple-domain. Among MCI patients, the most commonly affected cognitive domains were executive functions (85% of MCI patients), long-term memory (70%), and visuo-spatial abilities (46%). No significant differences emerged between patients and HC in demographic variables [Chi-squared test on sex variable: X ^2^_(2)_ = 1.15, *p* = 0.6; one-way ANOVA on age variable: *F*_(2,31.7)_ = 0.11, *p* = 0.9], apart from education [PD-MCI < PD-CU: one-way ANOVA *F*_(2,39.8)_ = 3.82, *p* = 0.03] (Table 2). At cognitive level, MCI patients showed lower performance than CU in long-term memory (RAVLT, *p* = 0.002; ROCF, *p* < 0.001), attention (attentive matrices, *p* < 0.001) executive functions (Stroop time interference effect, *p* = 0.002; Stroop error interference effect, *p* < 0.001; verbal fluency on phonemic cue, *p* < 0.001), language (naming of colored pictures, *p* = 0.005; naming of actions, *p* = 0.005) and visuo-spatial abilities (line orientation judgment test:, *p* = 0.003; unknown face recognition task, *p* = 0.004).

**TABLE 2:** Demographic, clinical, and neuropsychological features of Parkinson’s disease (PD) patients and healthy controls (HC).

Variable	PD-MCI (*n* = 13)	PD-CU (*n* = 32)	HC (*n* = 45)	Statistics	*Post-hoc*
**Demographics**					
Sex (M/F)	9/4	17/15	24/21	X ^2^_(2)_ = 1.15, *p* = 0.6	–
Age (years)	69 ± 8.8	67.9 ± 6.6	67.7 ± 7.7	*F*_(2,31.7)_ = 0.11, *p* = 0.9	–
Education (years)	9.5 ± 2.5	12.1 ± 4.1	11.5 ± 3.6	*F*_(2,39.8)_ = 3.82, *p* = 0.03	MCI < CU*
**Clinical**					
Disease duration (months)	84 [48–120]	84 [45.5–117]	–	U = 199, *p* = 0.9	–
LEDD	527.8 ± 176.3	571.6 ± 289.7	–	*t*_(39)_ = 0.50, *p* = 0.6	–
Hoehn & Yahr scale	2.5 [2–2.75]	2 [1–2]	–	U = 103, *p* = 0.1	–
Geriatric Depression Scale	6 [2.5–13.5]	11 [8–14]		U = 137, *p* = 0.1	–
Parkinson Anxiety Scale	14 ± 8.8	11.2 ± 7.4		*t*_(42)_ = 1.03, *p* = 0.3	–
**Neuropsychological**					
MoCA	18.9 ± 3.7	22.8 ± 2.9	23.9 ± 1.9	*F*_(2,84)_ = 18.29, ***p* < 0.001**	MCI < CU**; MCI < HC***
Digit backward	4 ± 0.7	4.3 ± 0.8	–	*t*_(43)_ = 1.02, *p* = 0.3	–
Attentive matrices	38.4 ± 6.7	48.2 ± 7.5	–	*t*_(43)_ = 4.06, ***p* < 0.001**	–
Rey auditory verbal learning delayed recall	6.7 ± 2.5	9.6 ± 2.6	–	*t*_(43)_ = 3.36, ***p* = 0.002**	–
Rey-Osterrieth complex figure delayed recall	9.5 ± 3.6	16.4 ± 5.9	–	*t*_(43)_ = 3.88, *p* < 0.001	–
Stroop time interference effect	25.6 [19.5–42]	13.3 [10–19]	–	U = 72.5, ***p* = 0.002**	–
Stroop error interference effect	5.2 [2.4–6.1]	0 [0–0.4]	–	U = 20, ***p* < 0.001**	–
Verbal fluency on phonemic cue	24.7 ± 8.8	36.2 ± 9.1	–	*t*_(43)_ = 3.88, ***p* < 0.001**	–
Naming (figures)	45.9 [44.7–47]	47.8 [46.6–48]	–	U = 99.5, ***p* = 0.005**	–
Naming (actions)	46 [43.9–48.5]	49.1 [47.4–50]	–	U = 85.5, ***p* = 0.005**	–
Line orientation judgment test	20.5 ± 6.1	25.1 ± 3.5	–	*t*_(43)_ = 3.15, ***p* = 0.003**	–
Unknown face recognition task	41.9 ± 5.0	46.6 ± 4.2	–	*t*_(42)_ = 3.09, ***p* = 0.004**	–

*Descriptive data are given as Mean ± Standard Deviation (SD) for parametric statistics; Median [25–75th percentile] for non-parametric statistics.*

*p–p-value.*

*p < 0.05, **p < 0.01, ***p < 0.001.

*Bold font—Significant p-values.*

*CU, cognitively unimpaired; MCI, mild cognitive impairment; HC, healthy controls; LEDD, L-dopa equivalent daily dose; MoCA, montreal cognitive assessment.*

### Socio-Cognitive Deficits in Parkinson’s Disease Patients Classified According to Cognitive Status

The analysis of social performance between PD-MCI, PD-CU, and HC showed significant differences in both emotion recognition and attribution tasks (Table 3). Global emotion recognition abilities were reduced in PD-MCI (*p* < 0.001) compared to PD-CU (*p* = 0.009) and HC (*p* = 0.003). In particular, PD-MCI patients showed lower scores than HC in the recognition of fear (*p* = 0.02), surprise (*p* = 0.002) and sadness (*p* < 0.001). Anger (*p* = 0.04) and sadness (*p* < 0.001) recognition was reduced in PD-MCI compared to PD-CU patients. Together with emotion recognition deficits, PD-MCI showed worse performance than HC in SET scores of emotion (SET-EA, *p* < 0.001) and intention (SET-IA, *p* < 0.001) attribution, as well as in the control condition of causal inference (SET-CI, p = 0.03). Notably, SET-EA (*p* = 0.002) and SET-IA (*p* = 0.02) were also reduced in PD-CU compared to HC, while only a trend emerged in SET-CI (*p* = 0.05).

**TABLE 3:** Performance comparison at social tasks between Parkinson’s disease (PD) patients characterized according to cognitive status and healthy controls (HC).

	HC	PD-CU	PD-MCI	Statistics	*Post-hoc*
Ek60 global score	50.7 ± 4.6	49.6 ± 5.7	42.4 ± 6.8	*F*_(2,86)_ = 12.21, ***p* < 0.001**	MCI < CU**; MCI < HC**
Surprise	10 [9–10]	9 [8–10]	8 [7–9]	H_2_ = 11.91, ***p* = 0.003**	MCI < HC**
Happiness	10 [10–10]	10 [10–10]	10 [9–10]	H_2_ = 2.96, *p* = 0.228	–
Fear	4 [2–7]	4 [2–5.5]	2 [0–3]	H_2_ = 7.3, ***p* = 0.03**	MCI < HC*
Disgust	9 [7–10]	8 [7–9.5]	7 [6–9]	H_2_ = 5.12, *p* = 0.08	–
Anger	7 [7–8]	7 [7–9]	6 [4–8]	H_2_ = 6.66, ***p* = 0.04**	MCI < CU*
Sadness	8 [7–9]	8 [8–9]	5 [4–6]	H_2_ = 18.65, ***p* < 0.001**	MCI < HC***; MCI < CU***
SET global score	17.1 [15.2–17.3]	15.3 [11.3–16.2]	11.2 [7.7–13.3]	H_2_ = 29.33, ***p* < 0 0.001**	MCI < CU*; CU < HC***; MCI < HC***
SET emotion attribution	6 [5.05–6]	4.9 [3.6–5.6]	3.1 [2.1–3.3]	H_2_ = 25.62, ***p* < 0 0.001**	MCI < CU*; CU < HC**; MCI < HC***
SET intention attribution	6 [5.2–6]	5.2 [4.05–6]	4 [3.1–4.2]	H_2_ = 24.86, ***p* < 0.001**	MCI < CU*; CU < HC*; MCI < HC***
SET causal inference	6 [5.1–6]	5.05 [3.9–6]	4.3 [3.4–5.2]	H_2_ = 9.43, ***p* = 0.01**	MCI < HC*

*Descriptive data are given as Mean ± Standard Deviation (SD) for parametric statistics; Median[25–75th percentile] for non-parametric statistics.*

*p–p-value.*

*p < 0.05, **p < 0.01, ***p < 0.001.

*Bold font—significant p-values; CU, cognitively unimpaired; MCI, mild cognitive impairment; HC, healthy controls; Ek60, Ekman 60-faces test; SET, story-based empathy task.*

Partial correlation (Spearman, r_*s*_) results are reported in Table 4. These analyses showed a significant association between Ek60 and SET-EA scores (r_*s*_ = 0.48, *p* < 0.004), while no significant correlations emerged with SET-IA or SET-CI sub-scores. Executive functions (i.e., Stroop task) were significantly associated to the performance at both SET (SET-EA: r_*s*_ = –0.43, *p* = 0.01, SET-IA: r_*s*_ = –0.45, *p* = 0.01, SET-CI: r_*s*_ = –0.41, *p* = 0.02) and Ek60 (r_*s*_ = –0.37, *p* = 0.02) tasks. SET-EA performance was also associated to language functions (naming of colored pictures: r_*s*_ = 0.52, *p* = 0.001; naming of actions: rs = 0.38, *p* = 0.03). Finally, scores at the Ek60 test were positively associated with the performance at the unknown face recognition task (r_*s*_ = 0.50, *p* < 0.001).

**TABLE 4:** Partial correlations between social tasks, cognitive, and behavioral variables.

	SET emotion attribution	SET intention attribution	SET causal inference	Ek60 global score
Ek60 global score	**r_*s*_ = 0.48 *p* = 0.004**	r_*s*_ = 0.32 *p* = 0.06	r_*s*_ = 0.14 *p* = 0.41	—
Digit backward	r_*s*_ = 0.26 *p* = 0.13	r_*s*_ = 0.13 *p* = 0.48	r_*s*_ = 0.09 *p* = 0.59	r_*s*_ = 0.02 *p* = 0.90
Attentive matrices	r_*s*_ = 0.32 *p* = 0.05	r_*s*_ = 0.30 *p* = 0.08	r_*s*_ = –0.09 *p* = 0.62	**r_*s*_ = 0.40 *p* = 0.007**
Rey auditory verbal learning delayed recall	r_*s*_ = 0.08 *p* = 0.66	r_*s*_ = 0.31 *p* = 0.07	r_*s*_ = –0.08 *p* = 0.64	r_*s*_ = 0.18 *p* = 0.24
Rey-Osterrieth complex figure recall	r_*s*_ = 0.28 *p* = 0.10	**r_*s*_ = 0.40 *p* = 0.02**	**r_*s*_ = 0.41 *p* = 0.01**	**r_*s*_ = 0.47 *p* = 0.002**
Verbal fluency on phonemic cue	r_*s*_ = 0.33 *p* = 0.05	r_*s*_ = 0.34 *p* = 0.05	r_*s*_ = 0.30 *p* = 0.08	**r_*s*_ = 0.60 *p* < 0.001**
Stroop time interference effect	**r_*s*_ = –0.43 *p* = 0.01**	**r_*s*_ = –0.45 *p* = 0.01**	**r_*s*_ = –0.41 *p* = 0.02**	**r_*s*_ = –0.37, *p* = 0.02**
Unknown face recognition task	r_*s*_ = 0.22 *p* = 0.20	r_*s*_ = 0.22 *p* = 0.20	r_*s*_ = 0.08 *p* = 0.64	**r_*s*_ = 0.50 *p* < 0.001**
Line orientation judgment task	r_*s*_ = 0.30 *p* = 0.07	r_*s*_ = 0.10 *p* = 0.55	r_*s*_ = 0.16 *p* = 0.34	r_*s*_ = 0.12 *p* = 0.44
Naming of colored pictures	**r_*s*_ = 0.52 *p* = 0.001**	r_*s*_ = 0.26 *p* = 0.13	r_*s*_ = 0.24 *p* = 0.16	**r_*s*_ = 0.51 *p* < 0.001**
Naming of actions	**r_*s*_ = 0.38 *p* = 0.03**	r_*s*_ = 0.08 *p* = 0.62	r_*s*_ = –0.08 *p* = 0.65	r_*s*_ = 0.28 *p* = 0.07
Parkinson Anxiety Scale	r_*s*_ = 0.07 *p* = 0.69	r_*s*_ = –0.03 *p* = 0.85	r_*s*_ = –0.17 *p* = 0.33	r_*s*_ = –0.06 *p* = 0.71
Geriatric Depression Scale	r_*s*_ = –0.10 *p* = 0.59	r_*s*_ = –0.26 *p* = 0.12	**r_*s*_** = –0.19 *p* = 0.28	r_*s*_ = –0.22 *p* = 0.15

*Descriptive data are given as Mean ± Standard Deviation (SD) for parametric statistics; Median [25–75th percentile] for non-parametric statistics.*

*r_s_, Spearman’s rank correlation coefficient.*

*p–p-value.*

*Bold font—significant p-values; Ek60, Ekman 60-faces test; SET, story-based empathy task.*

No significant correlations emerged between social cognitive performance and mood disturbances evaluated through GDS and PAS scales. Moreover, no significant correlations were found between LEDD, disease duration, Hoehn and Yahr stage and performances at social tasks in either the total group of PD patients or the CU and MCI subgroups.

### Socio-Cognitive Deficits in Parkinson’s Disease Patients According to Normative Data

**FIGURE 1:**
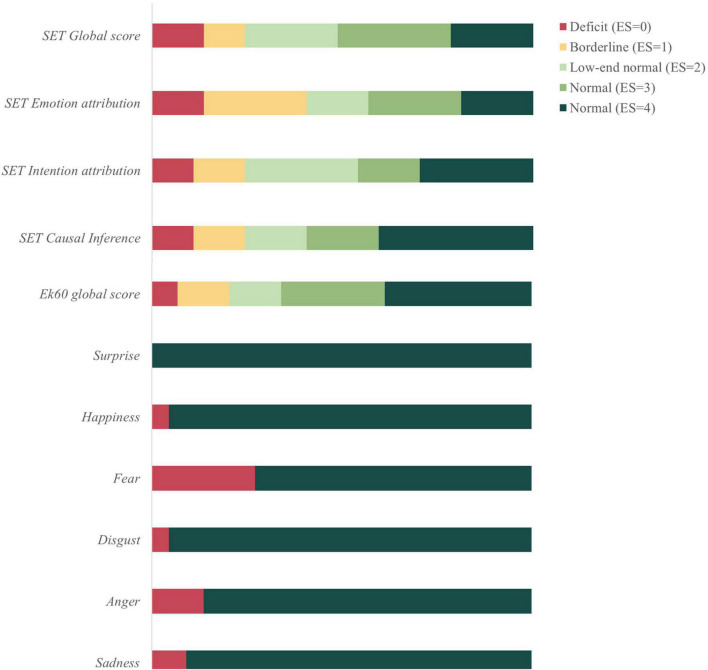
Socio-cognitive dysfunctions in Parkinson’s disease (PD) patients compared to healthy controls (HC). SET, story-based empathy task; Ek60, Ekman 60-faces test; ES, equivalent scores according to Capitani and Laiacona (1997) method.

PD patients’ performance at socio-cognitive tasks (i.e., SET and Ek60 test) was finally evaluated according to Italian normative data (Dodich et al., 2014, 2015) to define the prevalence of patients with socio-cognitive dysfunctions. In particular, nine patients showed poor performance in social tasks, five of them classified as PD-CU and four as MCI. Analyzing the socio-cognitive profile of these patients in more detail, two PD-MCI patients showed a deficit in both Ek60 and SET tasks, while seven patients (2 PD-MCI, 5 PD-CU) presented an isolated deficit at either the Ek60 (one patient) or SET task (six patients). Among patients showing a defective SET performance, four presented isolated deficits in mentalizing sub-tasks (i.e., SET-IA and/or SET-EA), while the others were characterized by an overall deficit (i.e., mental states attribution and control condition). A borderline performance in SET-EA, IA and CI was found in 10, 5, and 5 patients, respectively, while 6 patients showed a borderline performance at the Ek60 test. Exploratively, we also evaluated the performance at single emotions recognition. Fear represented the most difficult emotion to be recognized (impaired in 12 patients) followed by anger (*n* = 6) and sadness (*n* = 4) (see Figure 1 for details on socio-cognitive deficits in PD according to normative data).

Finally, when considering social cognition among the cognitive domains for MCI criteria, two patients originally classified as PD-CU were re-classified as PD-MCI multiple domains, presenting a significant impairment in two neuropsychological tasks (i.e., socio-cognitive and executive functions task).

## Discussion

Increasing evidence reports significant socio-cognitive dysfunctions in PD. However, the extent of these deficits according to the disease stage, as well as their clinical relevance, is still unclear. Thus, in this study, we adopted two social tasks clinically standardized for the Italian population in order to assess emotion recognition and ToM in PD, with the final aim of investigating the prevalence of socio-cognitive deficits in a sample of non-demented patients classified according to MDS Level II criteria for MCI (Litvan et al., 2012).

Emotion recognition and ToM represent two core components of social cognition, driving social interaction through automatic and voluntary processes (Coricelli, 2005). Notably, these cognitive functions require the integrity of a set of specific and shared brain regions belonging to frontal and mesocorticolimbic circuits (Mier et al., 2010; Abu-Akel and Shamay-Tsoory, 2011), which can be significantly affected by PD neurodegenerative processes (Baggio et al., 2012; Diederich et al., 2016). In support of this evidence, we found significant deficits in both facial emotion recognition and mental states attribution in PD-MCI. Notably, despite previous authors suggested a progressive impairment in cognitive ToM and a later involvement of affective ToM with disease progression [e.g., Poletti and Bonuccelli (2012)], our results support the presence of early alterations in both cognitive and affective facets of mental states attribution (Santangelo et al., 2012; Enrici et al., 2015; Coundouris et al., 2020). This evidence is further supported by the results on PD-CU group, which showed an isolated deficit in SET-IA and SET-EA.

Together with mentalizing deficits, PD-MCI showed a significant impairment in negative emotion recognition in agreement with previous quantitative and qualitative literature reviews (Gray and Tickle-Degnen, 2010; Argaud et al., 2018). Notably, fear represented the most difficult emotion to recognize, confirming previous evidence [e.g., Mattavelli et al. (2021)], followed by anger and sadness. On the other hand, no significant deficit has been found in disgust recognition. This result is inconsistent with earlier studies reporting a disproportionate deficit in disgust recognition in PD [e.g., Suzuki et al. (2006)] ascribable to the disruption of the basal ganglia–insula system (Obeso et al., 2008) involved in the recognition of this emotion (Phan et al., 2002; Fusar-Poli et al., 2009). However, more recent quantitative approaches showed heterogeneous results when considering single emotions, and a deficit in disgust recognition was found only in 47% of the studies taken into consideration (Argaud et al., 2018). Many potential confounding factors, such as disease severity, medication, or mood disorders (Gray and Tickle-Degnen, 2010) could contribute to emotion recognition deficit in PD, causing high variability in study results. Despite we did not find a significant relationship in our sample with dopaminergic treatment or mood disorders, future studies should be devoted to fully elucidate the role of these factors in PD emotion recognition deficits.

Meta-analytical evidence concurs however in reporting a major impairment in the recognition of negative emotions rather than of positive ones, and this deficit has been previously associated with amygdalar (Diederich et al., 2016) and mesocorticolimbic alterations (Ibarretxe-Bilbao et al., 2009; Baggio et al., 2012) in PD. In this sense, it is interesting to underline that in the current study we found a specific correlation between emotion recognition abilities and affective ToM, evaluated through SET-EA. Considering that a low performance at this sub-task has been previously related to amygdalar structural damage in other neurological populations (Cerami et al., 2014; Campanella et al., 2021), these results suggest possible common underlying pathological mechanisms affecting both emotion recognition and attribution in these patients. This perspective opens new relevant research questions that should be further explored. Indeed, although recent models have underlined the role of these socio-cognitive facets in social interaction [e.g., Cassel et al. (2019)], it is still to be fully elucidated the role of socio-cognitive deficits (in terms of emotion recognition or ToM) in altering social behavior.

Despite the significant results at group level, when evaluating socio-cognitive performance in single subjects according to normative data we found a limited number of patients showing a deficitary performance. This possibly suggests in the PD group the presence of subtle alterations in socio-cognitive tasks, still below the threshold of clinical relevance. This hypothesis is further supported by the presence of patients showing a borderline performance in both emotion recognition and mental states attribution. When considering normative cut-off scores, 20% of patients showed a significant clinical deficit in global emotion recognition or mental state processing. This percentage is similar to what has been previously reported (i.e., 30%) (Czernecki et al., 2021). The mismatch could be possibly explained in light of the different criteria adopted to define MCI [i.e., MDS Level II criteria in the current study and MDS Level I criteria in Czernecki et al. (2021)]. Indeed, MDS Level I criteria require an abbreviated evaluation compared to Level II criteria, with a foreseeable effect on diagnostic certainty, extent of clinical characterization and MCI detectability (Litvan et al., 2012). In accordance with this consideration, we found a higher percentage of patients characterized by MCI (i.e., 29%) compared to Czernecki’s study (i.e., 15.6%) (Czernecki et al., 2021), and, consistently, a lower percentage of PD-CU (i.e., 11%) showing socio-cognitive deficits.

When we included social cognition among the cognitive domains for MCI criteria, two PD-CU patients were re-classified as PD-MCI multiple domains. Considering the contribution of MCI classification in predicting the hazard of PD dementia (Hoogland et al., 2017), this result indicates a possible benefit in considering social cognition among the MDS cognitive domains in order to improve MCI detectability. Consistently with previous literature findings (Litvan et al., 2012; Barvas et al., 2021), the classification of our PD sample according to MDS Level II criteria showed predominant executive functioning, memory, and visuospatial deficits, but none of the patients satisfied the criteria for PD-MCI single domain. This result is in line with previous evidence showing a prevalence of multiple domain impairments (Goldman et al., 2015), but suggests potential challenges in identifying domain-specific PD-MCI subtypes using MDS criteria.

The main limitations of this study are represented by the small sample size that might affect the statistical power of the analyses, and by the lack of a detailed neuropsychological characterization of healthy controls, which does not allow to exclude the presence in this sample of subtle deficits in single cognitive domains. Despite the MoCA cut-off score used to include healthy participants is lower than the one suggested by international meta-analytic results [e.g., Carson et al. (2018)], this is in line with normative data provided by other Italian studies (Santangelo et al., 2015; Aiello et al., 2022). Besides, due to the limited availability of validated socio-cognitive tests, other sub-components of social cognition were not assessed. Finally, no motor scores including hypomimia were included in this study, thus preventing to evaluate the role of reduced facial mimicry in social tasks, particularly for emotion recognition (Künecke et al., 2014; Prenger and Macdonald, 2018).

In conclusion, the results of the present study support that alterations in affective recognition and attribution may occur in PD from the earliest stages of the disease. In agreement with previous evidence (Czernecki et al., 2021), we highlighted the presence of a PD subgroup with socio-cognitive dysfunctions, which in a small percentage of patients represented an isolated deficit, overall supporting the importance of including social cognition in PD neuropsychological assessment. The relevance of socio-cognitive evaluation in clinical practice has been recently underlined in different neurological populations (Henry et al., 2016; Cotter et al., 2018; Dodich et al., 2020), also in consideration of the significant consequences of these deficits in social integration, well-being and quality of life (Bodden et al., 2010; Martinez et al., 2018; Dodich et al., 2021b). The inclusion of social tasks in the cognitive assessment of PD, as well as in the evaluation of MCI due to other neurodegenerative diseases (e.g., Alzheimer’s Disease) (Boccardi et al., 2021), will promote the full characterization of these deficits, as well as their clinical role in a diagnostic and prognostic framework.

## Data Availability Statement

The raw data supporting the conclusions of this article will be made available by the authors, without undue reservation.

## Ethics Statement

The studies involving human participants were reviewed and approved by University of Trento Ethical Committee. The patients/participants provided their written informed consent to participate in this study.

## Author Contributions

AD, CP, LT, and FZ: conception and organization of the project. MP, CL, MM, and RD: data acquisition. AD, CM, and GF: data interpretation and data analyses. AD and GF: first drafting of the work. All authors revised the manuscript and provided the approval of the work.

## Conflict of Interest

The authors declare that the research was conducted in the absence of any commercial or financial relationships that could be construed as a potential conflict of interest.

## Publisher’s Note

All claims expressed in this article are solely those of the authors and do not necessarily represent those of their affiliated organizations, or those of the publisher, the editors and the reviewers. Any product that may be evaluated in this article, or claim that may be made by its manufacturer, is not guaranteed or endorsed by the publisher.
